# Polyfluoroalkyl Chemicals in the U.S. Population: Data from the National Health and Nutrition Examination Survey (NHANES) 2003–2004 and Comparisons with NHANES 1999–2000

**DOI:** 10.1289/ehp.10598

**Published:** 2007-08-29

**Authors:** Antonia M. Calafat, Lee-Yang Wong, Zsuzsanna Kuklenyik, John A. Reidy, Larry L. Needham

**Affiliations:** Division of Laboratory Sciences, National Center for Environmental Health, Centers for Disease Control and Prevention, Atlanta, Georgia, USA

**Keywords:** biomonitoring, C8, exposure, PFCs, PFOA, PFOS, prevalence, serum

## Abstract

**Background:**

Polyfluoroalkyl chemicals (PFCs) have been used since the 1950s in numerous commercial applications. Exposure of the general U.S. population to PFCs is widespread. Since 2002, the manufacturing practices for PFCs in the United States have changed considerably.

**Objectives:**

We aimed to assess exposure to perfluorooctane sulfonic acid (PFOS), perfluorooctanoic acid (PFOA), perfluorohexane sulfonic acid (PFHxS), perfluorononanoic acid (PFNA), and eight other PFCs in a representative 2003–2004 sample of the general U.S. population ≥ 12 years of age and to determine whether serum concentrations have changed since the 1999–2000 National Health and Nutrition Examination Survey (NHANES).

**Methods:**

By using automated solid-phase extraction coupled to isotope dilution–high-performance liquid chromatography–tandem mass spectrometry, we analyzed 2,094 serum samples collected from NHANES 2003–2004 participants.

**Results:**

We detected PFOS, PFOA, PFHxS, and PFNA in > 98% of the samples. Concentrations differed by race/ethnicity and sex. Geometric mean concentrations were significantly lower (approximately 32% for PFOS, 25% for PFOA, 10% for PFHxS) and higher (100%, PFNA) than the concentrations reported in NHANES 1999–2000 (*p* < 0.001).

**Conclusions:**

In the general U.S. population in 2003–2004, PFOS, PFOA, PFHxS, and PFNA serum concentrations were measurable in each demographic population group studied. Geometric mean concentrations of PFOS, PFOA, and PFHxS in 2003–2004 were lower than in 1999–2000. The apparent reductions in concentrations of PFOS, PFOA, and PFHxS most likely are related to discontinuation in 2002 of industrial production by electrochemical fluorination of PFOS and related perfluorooctanesulfonyl fluoride compounds.

Concern about exposure of the ecosystem, including humans, to halogenated persistent organic pollutants (POPs) has existed for several decades. Many of these chemicals are persistent and toxic, tend to bioaccumulate, and can undergo long range atmospheric transport; for these reasons, their production has been banned or reduced worldwide, leading to their decreased concentrations in the ecosystem. In addition, adherence to provisions set forth in the Stockholm Convention on POPs for 12 organochlorine chemicals (United Nations Environment Programme 2004) probably will result in continued decreasing environmental concentrations. More recently, the focus of environmental and public health concern has shifted from chlorinated chemicals to brominated and fluorinated chemicals.

Among the fluorinated chemicals, the polyfluoroalkyl chemicals (PFCs) have been used extensively since the 1950s in commercial applications, including surfactants, lubricants, paper and textile coatings, polishes, food packaging, and fire-retarding foams. Some of these PFCs, including perfluorooctane sulfonic acid (PFOS) and perfluorooctanoic acid (PFOA), persist in humans and the environment and have been detected worldwide in wildlife (Houde et al. 2006 and references therein). Exposure to PFOS and PFOA in the general population also is widespread, although demographic, geographic, and temporal differences may exist (Calafat et al. 2006b, 2007; Fromme et al. 2007; Guruge et al. 2005; Hansen et al. 2001; Harada et al. 2007; Kannan et al. 2004; Karrman et al. 2006; Olsen et al. 2005; Taniyasu et al. 2003; Yeung et al. 2006).

No definite association has been established between exposure to PFOS and PFOA and adverse health effects in several occupational studies (Alexander et al. 2003; Gilliland and Mandel 1993; Grice et al. 2007; Olsen et al. 2004a) and in one population exposed to PFOA through contaminated drinking water (Emmett et al. 2006). Negative associations between cord serum concentrations of both PFOS and PFOA and birth weight and ponderal index, but not newborn length or gestational age, have been reported in a nonoccupational population (Apelberg et al. 2007). By contrast, no association has been reported between employment in jobs with high exposure to PFOS before the end of pregnancy and maternally reported birth weight (Grice et al. 2007). In animals, exposure to PFOS and PFOA is associated with adverse health effects (Kennedy et al. 2004; Lau et al. 2004; Organisation for Economic Co-operation and Development 2002) albeit at serum concentrations orders of magnitude higher than the concentrations observed in the general population (Butenhoff et al. 2004; Luebker et al. 2005). Because of these compounds’ known toxicity to animals, their ubiquitous presence, and their persistence in humans, wildlife, and the environment, PFCs research is of interest to toxicologists, epidemiologists, and environmental and public health scientists.

Biomonitoring data for these PFCs in the general population are needed to assess current exposures and to determine whether technologic changes affect human exposures to these compounds. As part of the continuous U.S. National Health and Nutrition Examination Survey (NHANES), urine and serum samples are collected and analyzed for selected environmental chemicals [Centers for Disease Control and Prevention (CDC) 2005]. NHANES participants also provide sociodemographic information and medical history and undergo standardized physical examinations (CDC 2003). We recently reported the concentrations of PFOS, PFOA, and nine other PFCs in 1,562 participants from NHANES 1999–2000 (Calafat et al. 2007). The high frequency of detection of PFOS and PFOA suggested highly prevalent exposures to these compounds at a time when both were being manufactured in the United States. In 2002, the 3M Company (St. Paul, MN), the sole U.S. producer of PFOS, discontinued its production of PFOS and related perfluorooctanesulfonyl fluoride (POSF)–based chemistries by electrochemical fluorination. Although PFOA and its salts and precursors still are manufactured by others by a different process, reductions in their manufacturing emissions have been proposed [Prevedouros et al. 2006; U.S. Environmental Protection Agency (EPA) 2006]. We now report the serum concentrations of 12 PFCs, including PFOS and PFOA, in 2,094 participants from NHANES 2003–2004 and compare these data with data from NHANES 1999–2000 (Calafat et al. 2007). The 2003–2004 data provide the first estimates of serum PFC concentrations in a representative U.S. population since implementation of the changes in manufacturing practices for some PFCs in the United States.

## Materials and Methods

We obtained serum samples analyzed for PFCs from 2,094 participants ≥ 12 years of age from NHANES 2003–2004. The National Centers for Health Statistics Institutional Review Board reviewed and approved the study protocol. All participants provided informed written consent; parents or guardians provided consent for participants < 18 years of age (CDC 2006a).

We measured perfluorooctane sulfonamide (PFOSA), 2-(*N*-ethyl-perfluorooctane sulfon-amido) acetic acid (Et-PFOSA-AcOH), 2-(*N*-methyl-perfluorooctane sulfonamido) acetic acid (Me-PFOSA-AcOH), perfluorobutane sulfonic acid (PFBuS), perfluorohexane sulfonic acid (PFHxS), PFOS, PFOA, perfluoroheptanoic acid (PFHpA), perfluorononanoic acid (PFNA), perfluorodecanoic acid (PFDeA), perfluoroundecanoic acid (PFUA), and perfluorododecanoic acid (PFDoA) in 1 mL of serum, using a modification of the method of Kuklenyik et al. (2004), which involved automated solid-phase extraction coupled to reversed-phase high-performance liquid chromatography–tandem mass spectrometry. We used ^18^O_2_-PFOS (for all sulfonic acids and all amides) and ^13^C_2_-PFOA (for all carboxylic acids) for quantification. To compensate for the lack of isotope-labeled internal standards for the other analytes and to partially account for matrix effects, the calibration standards were spiked into calf serum. The limits of detection (LODs) ranged from 0.1 to 1.0 μg/L; the accuracy ranged from 84 to 135% at three concentrations (Kuklenyik et al. 2004); and the precision ranged from around 10 to 26% at two different levels (Table 1). Low-concentration (~ 3 μg/L to ~ 9 μg/L) and high-concentration (~ 10 μg/L to ~ 30 μg/L) quality-control (QC) materials, prepared from a base calf serum pool, were analyzed with reagent blank, serum blank, and NHANES samples (Kuklenyik et al. 2004). Standard, blank, QC, and NHANES samples were analyzed by the procedure described above.

We analyzed the data using SAS (version 9.1.3; SAS Institute Inc., Cary, NC) and SUDAAN (version 9.0.1; Research Triangle Institute, Research Triangle Park, NC). SUDAAN calculates variance estimates after incorporating the sample population weights, designed for the one-third subset of the full survey, which account for unequal selection probabilities and planned oversampling of certain subgroups resulting from the complex multistage area probability design of NHANES. Race/ethnicity was defined on the basis of self-reported data as non-Hispanic black, non-Hispanic white, and Mexican American. Persons not defined by these groups were included only in the total population estimate. Age was reported in years at the most recent birthday. We estimated the weighted percentage of detection and calculated weighted geometric means and percentiles for the serum concentrations (in micrograms per liter) of the various PFCs. For concentrations below the LOD, as recommended for the analysis of NHANES data (CDC 2006b), we used a value equal to the LOD divided by the square root of 2 (Hornung and Reed 1990). Parametric statistics were computed only for analytes for which the frequency of detection was ≥ 60%. Because PFC concentrations were not normally distributed, we used the natural log transformation. Weighted Pearson correlation coefficients and related *p-*values were calculated in SAS. Statistical significance was set at *p* < 0.05.

We used analysis of covariance to examine the influence of demographic and socioeconomic variables on the log-transformed serum concentrations of PFOS, PFOA, PFHxS, and PFNA. For multiple regression, we calculated the least square geometric means (LSGM) and compared them for each categorical variable. The variables included in the initial model were as follows: age as a continuous variable, sex, race/ethnicity, smoking status (yes/no), and education (less than high school, high school diploma, more than high school). Participants were categorized as smokers if their serum cotinine concentrations were > 10 μg/L. We chose to include education in the model without household income to minimize the possibility of collinearity because *a*) income and education are strongly associated (chi-square *p* = 0.001) and *b*) the final model yielded comparable results with either variable separately (except for PFOS, which included one additional significant term between income and smoking status). We assessed all possible two-way interaction terms in the model.

To reach the final reduced model, we used backward elimination with a threshold of *p* < 0.05 for retaining the variable in the model, using Satterwaite adjusted *F* statistics. We evaluated for potential confounding by adding each of the excluded variables back into the final model one by one and examining changes in the β coefficients of the statistically significant main effect. If addition of one of these excluded variables caused a change in a β coefficient by ≥ 10%, the variable was re-added to the model.

## Results

The distribution of PFC serum concentrations is reported stratified by age, sex, and race/ethnicity (Tables 2–5). Four analytes were detected in > 98% of the samples (PFOS, 99.9%; PFOA, 99.7%; PFHxS, 98.3%; PFNA, 98.8%). Concentrations of these four PFCs ranged from < 0.4 μg/L to 435 μg/L (PFOS), < 0.1 μg/L to 77.2 μg/L (PFOA), < 0.3 μg/L to 82.0 μg/L (PFHxS), and < 0.1 μg/L to 11.5 μg/L (PFNA). Six other analytes were detected at lower frequencies: PFDeA (31.3%), Me-PFOSA-AcOH (27.5%), PFOSA (22.2%), PFUA (9.7%), PFHpA (6.2%), and Et-PFOSA-AcOH, (3.4%); their geometric mean and selected percentile concentrations are given as Supplemental Material in Tables S1–S6 (online at http://www.ehponline.org/docs/2007/10598/suppl.pdf). For the two analytes detected in < 1% of the samples (PFDoA, < 0.1%; PFBuS, 0.4%), we could not calculate the 95th percentile of concentrations.

Statistically significant correlations (*p <* 0.001) existed between the log-transformed concentrations of PFOS and PFOA (Pearson correlation coefficient *r* = 0.66), PFHxS (*r* = 0.56), and PFNA (*r* = 0.50); between PFOA and PFHxS (*r* = 0.46) and PFNA (*r* = 0.55); and between PFHxS and PFNA (*r* = 0.17).

The final models included sex (*p* < 0.01), age, race/ethnicity, and age-by-race/ethnicity interaction (*p* = 0.01) for PFOS; sex, race/ethnicity, age, education, sex-by-age (*p* < 0.01), sex-by-race/ethnicity (*p* = 0.03), and education-by-age (*p* = 0.04) interactions for PFOA; sex, race/ethnicity (*p* = 0.01), age, and sex-by-age interaction (*p* = 0.02) for PFHxS; and sex (*p* < 0.01), race/ethnicity, age, education (*p* = 0.02), smoking status (*p* = 0.02), and race/ethnicity-by-age (*p* < 0.01) and age-by-smoking status (*p* = 0.04) interactions for PFNA. Because of these interactions with age, concentrations were compared at the 25th (age = 26 years), 50th (age = 41 years), 75th (age = 55 years), and 90th (age = 70 years) percentiles of age.

LSGM concentrations provide geometric mean estimates for a demographic variable after adjustment for the model covariates (Table 6). The statistical significance values when comparing these LSGM concentrations are shown in the Supplemental Material, Table S7 (online at http://www.ehponline.org/docs/2007/10598/suppl.pdf). PFOS LSGM concentrations were significantly higher (*p* < 0.01) in males than in females. Similarly, for PFOA and PFHxS, males had significantly higher LSGM concentrations than females except at the 90th percentile of age (Table 6). LSGM concentrations of PFHxS were significantly lower for Mexican Americans than for non-Hispanic blacks (*p* = 0.01) and non-Hispanic whites (*p* < 0.01); LSGM concentrations did not differ significantly between non-Hispanic whites and non-Hispanic blacks (*p* = 0.49). PFOS and PFNA LSGM concentrations were significantly lower in Mexican Americans than in non-Hispanic blacks (PFOS, *p* < 0.01; PFNA, *p* < 0.01–0.03) and non-Hispanic whites (PFOS, *p* < 0.01; PFNA, *p* < 0.01–0.02), regardless of age; LSGM concentrations between non-Hispanic whites and non-Hispanic blacks differed significantly only at the 75th and 90th percentiles of age (Table 6). Non-Hispanic whites had significantly higher PFOA LSGM concentrations (*p* < 0.01), regardless of sex, than Mexican Americans. The differences between Mexican-American males and non-Hispanic black males and between non-Hispanic white males and non-Hispanic black males were not statistically significant.

We used a two-sample *t*-test to compare the difference of the two geometric mean concentrations (on the log scale) of PFOS, PFOA, PFHxS, and PFNA during NHANES 1999–2000 and NHANES 2003–2004 (Table 7), taking into account their associated standard errors and degrees of freedom, by age, sex, and race/ethnicity, using SAS. The differences were all statistically significant (*p* < 0.05), except for PFHxS in Mexican Americans (*p* = 0.21) (Table 7). We analyzed the NHANES 2003–2004 samples first and then the NHANES 1999–2000 samples (Calafat et al. 2007) using two methods that differed in the manner in which PFCs were extracted and preconcentrated from the serum (Kuklenyik et al. 2004, 2005). In both methods, we used tandem mass spectrometry with ^18^O_2_-PFOS, ^13^C_2_-PFOA, and ^18^O_2_-PFOSA (only for NHANES 1999–2000) for quantification, the same multiple reaction monitoring transitions for quantification for PFOA (413/369) and PFOS (499/99), the same QC materials and analytical standards. ^18^O_2_-PFOSA was not commercially available when the 2003–2004 NHANES samples were analyzed. Except for PFNA and PFOA, for which the LODs were the same regardless of the method, the method used for NHANES 1999–2000 (Kuklenyik et al. 2005) had slightly lower LODs than the method used for NHANES 2003–2004 (Kuklenyik et al. 2004) (Table 1). To estimate whether method differences could account for the differences in concentrations, we analyzed QC samples from low and high concentration pools and 124 split samples using both methods. The two methods showed good agreement from the results of the split sample analysis [presented for PFOA in Figures S1 and S2 in Supplemental Material (online at http://www.ehponline.org/docs/2007/10598/suppl.pdf)]. Results were similar for all other analytes (data not shown). In general, analysis of the QC pools showed mean concentrations and coefficients of variation which were similar between the two methods (Table 1).

## Discussion

We detected PFOS, PFOA, PFHxS, and PFNA in > 98% of persons in this representative sample of the civilian, noninstitutionalized U.S. population, ≥ 12 years of age. These findings confirm that measurable serum concentrations of these compounds were prevalent in the United States in 2003–2004, even after 3M in 2002 discontinued its industrial production of PFOS and related compounds, including the ammonium salt of PFOA. Direct and indirect sources of PFOA still exist in the United States, although since 1999, global emissions of PFOA reportedly have decreased by more than half as of 2004 (Prevedouros et al. 2006), and current producers have committed to reducing manufacturing emissions of PFOA and its salts and precursors (U.S. EPA 2006).

Other PFCs, however, were detected infrequently. For example, PFBuS was detected in < 0.5% of the samples. PFBuS is a final degradation product of perfluorobutane-sulfonyl fluoride, now used in the manufacture of materials as a replacement for POSF-related chemicals [C-6 (e.g., PFHxS) and C-8 (e.g., PFOS)] that were phased out beginning in 2000. Similarly, in a study involving 18 volunteer employees from 3M Company, PFBuS was detected only in workers with production-related duties, whereas PFOA, PFOS, and PFHxS were detected in most workers (Ehresman et al. 2007). The lower frequency of detection of PFBuS than PFOS, PFOA, and PFHxS suggests that human exposures to PFBuS are indeed lower, and/or that pharmacokinetic factors, which might include increased urinary elimination, are different.

PFOS showed the highest geometric mean and 95th percentile concentrations, followed by PFOA, PFHxS, and PFNA. For PFOS, PFOA, and PFNA, however—unlike lipophilic POPs whose serum concentrations increase with age (Needham et al. 2006)—concentrations were quite similar among the four age groups (Tables 2–5), a finding that agrees with previous data (Calafat et al. 2007; Olsen et al. 2003, 2004b, 2004c). By contrast, for PFHxS, the geometric mean and 95th percentile concentrations were higher for adolescents than for adults, as previously reported (Calafat et al. 2007; Olsen et al. 2004b). The higher concentrations of PFHxS in children and adolescents could be related to their increased contact with carpeted floors containing PFHxS, which is used for specific postmarket carpet-treatment applications (Olsen et al. 2004b).

In agreement with previous reports (Calafat et al. 2006a, 2007; Fromme et al. 2007; Harada et al. 2004; Midasch et al. 2006; Yeung et al. 2006), we observed sex and race/ethnicity differences. Females had significantly lower LSGM concentrations of PFOS than did males (Table 6). For PFOA and PFHxS, sex differences also existed but were not as pronounced for the elderly (Table 6). Mexican Americans had the lowest LSGM concentrations of PFHxS and non-Hispanic whites and non-Hispanic blacks had similar concentrations (Table 6). Racial differences for PFOS and PFNA were age dependent, whereas those for PFOA were sex dependent (Table 6). These sex and racial differences may reflect variability in exposure patterns as a result of differences in factors such as lifestyle, diet, and use of products containing PFCs that may contribute to the observed serum concentrations of PFCs.

To evaluate whether the discontinued production of PFOS and related compounds by 3M Company in 2002 and technologic changes implemented by other companies have led to a subsequent decrease in serum PFC concentrations in the general U.S. population (Olsen et al. 2007b), we compared NHANES data of 1999–2000 with NHANES data of 2003–2004. The distribution of serum concentrations of PFOS, PFOA, PFHxS, and PFNA by sex, race/ethnicity, and age in 2003–2004 (Tables 2–5) was similar to that for the general U.S. population in 1999–2000 (Calafat et al. 2007). However, the geometric mean concentrations for PFOS, PFOA, and PFHxS in 2003–2004 were lower than for 1999–2000. For PFNA, 2003–2004 levels were higher than those found in 1999–2000. These concentrations differed significantly for all demographic groups except for PFHxS in Mexican Americans (Table 7). Various concentration percentiles similarly decreased for PFOS, PFOA, and PFHxS. We analyzed the NHANES 1999–2000 and 2003–2004 samples by using two different methods; however, these approaches provided equivalent results [Table 1; Figures S1 and S2 in the Supplemental Material (online at http://www.ehponline.org/docs/2007/10598/suppl.pdf)], indicating that the differences cannot be attributed to changes in the analytical methodology. The decrease in serum concentrations of PFOS and PFOA during this time interval agreed with the reported reductions in PFOS and PFOA concentrations for a group of Red Cross blood donors in the United States (Olsen et al. 2007b) and in PFOS (temporal trends for PFOA were not examined) in Arctic ringed seals in the same time (Butt et al. 2007). These decreases in serum concentrations of PFOS and PFOA in humans and wildlife had been related to the phaseout of POSF-based materials in 2000–2002 (Butt et al. 2007; Olsen et al. 2007b).

For PFHxS, although the geometric mean concentrations were lower in 2003–2004 than in 1999–2000, the differences were less evident, and in some cases they reversed at the higher concentration percentiles for some demographic categories. These findings may be related to the lower concentrations of PFHxS than of PFOS or PFOA and to differences in the estimated geometric mean serum elimination half-life (PFHxS, 7.3 years; PFOA, 3.5 years; and PFOS, 4.8 years) (Olsen et al. 2007a). Furthermore, the correlation between the serum concentrations of PFOS and PFOA was higher than correlations of PFHxS and either PFOA or PFOS, suggesting potential common exposure path-way(s) for PFOA and PFOS, but probably not for PFHxS (mostly used in carpet-treatment applications (Olsen et al. 2004b). Pharmacokinetic factors may also contribute to these differences. The transformation of certain POFS-related sulfonamides to PFOS and potentially to PFOA in the atmosphere was suggested as a common mechanism for formation of both PFOS and PFOA, which would account at least partly for the high correlation in serum concentrations (Olsen et al. 2007b). On the other hand, PFOA and other perfluorocarboxylates (e.g., PFNA), but not PFOS, might be formed from the biodegradation of the volatile fluorotelomer alcohols (Ellis et al. 2004).

Current manufacturing practices exclusively use fluorotelomers for the synthesis of perfluorocarboxylates (Prevedouros et al. 2006). Perfluorocarboxylates, including PFNA, were present as reaction by-products in POSF-based materials (Prevedouros et al. 2006). Interestingly, our data suggest that PFNA geometric mean concentrations in 2003–2004 approximately doubled over those of 1999–2000. However, because human exposure data for PFNA are more limited than they are for PFOS, PFOA, and even PFHxS, these results must be interpreted with caution. In 2004, the estimated annual production of the ammonium salt of PFNA, primarily used as a processing aid in the manufacture of such fluoropolymers as polyvinylidene fluoride, was 15–75 tonnes (Prevedouros et al. 2006). Information about efforts to reduce manufacturing emissions for PFNA, estimated at about 10% of the amount produced, was not found (Prevedouros et al. 2006). As a comparison, global manufacturing emissions of PFOA were about 15 tonnes in 2004, down from about 45 tonnes in 1999 (Prevedouros et al. 2006).

For most PFCs, these NHANES 2003–2004 results are consistent with reduced population exposure because of recent efforts of industry and government. U.S. and worldwide efforts continue in attempts to reduce exposures to PFCs, including PFOS and PFOA, and many halogenated POPs, including polybrominated diphenyl ethers. We will continue to assess exposure to these and other chemicals in the U.S. population through NHANES, an effort that will provide unique information on trends of exposure to these chemicals over time. In addition, we are analyzing pooled serum samples from 3- to 11-year-old children to fill data gaps for mean PFC concentrations in this age range.

## Figures and Tables

**Table 1:** LOD and precision data for the 12 polyfluoroalkyl compounds included in this study and a comparison of these parameters to the previously reported data for NHANES 1999–2000.

			Precision^a^			
	LOD (μg/L)^b^		QCL		QCH	
Analyte	NHANES 2003–2004	NHANES 1999–2000	NHANES 2003–2004	NHANES 1999–2000	NHANES 2003–2004	NHANES 1999–2000
PFOSA	0.2	0.05	2.7 (14.9)	2.4 (14.1)	13.0 (16.3)	12.4 (12.5)
Me-PFOSA-AcOH	0.6	0.2	3.4 (15.5)	3.1 (14.2)	9.1 (16.7)	9.0 (13.5)
Et-PFOSA-AcOH	0.4	0.2	3.8 (17.2)	3.5 (14.3)	8.3 (19.2)	8.1 (15.6)
PFBuS	0.4	ND	4.4 (18.2)	ND	14.6 (15.1)	ND
PFHxS	0.3	0.1	2.5 (16.4)	2.1 (16.6)	11.9 (12.9)	11.2 (12.3)
PFOS	0.4	0.2	8.9 (10.4)	8.8 (8.4)	31.4 (10.1)	31.6 (7.1)
PFHpA	0.3	0.4	7.6 (17.0)	6.8 (13.5)	15.8 (14.3)	15.5 (12.0)
PFOA	0.1	0.1	3.2 (10.0)	3.1 (8.5)	14.7 (10.9)	15.1 (7.3)
PFNA	0.1	0.1	2.5 (15.0)	2.6 (15.4)	12.7 (13.2)	13.0 (10.9)
PFDeA	0.3	0.2	2.4 (17.5)	2.2 (13.9)	8.5 (18.2)	8.4 (13.1)
PFUA	0.3	0.2	1.9 (22.0)	2.0 (19.1)	9.9 (19.8)	10.6 (16.2)
PFDoA	1.0	0.2	2.2 (25.6)	2.4 (22.4)	8.5 (25.7)	9.1 (19.3)

ND, not determined.

a Mean concentration (% coefficient of variation) of repeated measurements (minimum of 20) over time of quality-control calf serum materials of low (QCL) and high (QCH) concentrations.

b The NHANES 1999–2000 samples were analyzed by using the approach described in Kuklenyik et al. (2005), whereas the NHANES 2003–2004 samples were analyzed by using the Kuklenyik et al. (2004) approach.

**Table 2:** Geometric mean and selected percentiles (95% confidence intervals) of perfluorooctanesulfonate (PFOS) concentrations in serum (μg/L) for the U.S. population 12 years of age and older: data from NHANES 2003–2004.

Variable	Geometric mean	10th percentile	25th percentile	50th percentile	75th percentile	90th percentile	95th percentile	No.
All	20.7 (19.2–22.3)	9.8 (9.0–10.8)	14.6 (13.8–15.2)	21.1 (19.8–22.4)	29.9 (27.5–32.8)	41.2 (35.5–48.9)	54.6 (44.0–65.9)	2,094
12–19 years	19.3 (17.5–21.4)	9.9 (9.5–10.9)	14.4 (12.5–15.7)	19.9 (17.6–21.9)	27.1 (23.6–30.2)	36.5 (28.6–45.6)	42.2 (35.1–52.1)	640
20–39 years	18.7 (17.3–20.1)	8.9 (8.2–10.2)	12.6 (11.2–14.2)	18.7 (17.7–20.4)	27.4 (24.9–29.7)	36.9 (33.6–41.3)	44.3 (38.6–60.8)	490
40–59 years	22.0 (19.7–24.5)	10.6 (9.2–12.3)	15.3 (14.1–18.0)	22.2 (20.2–24.2)	32.2 (27.4–35.4)	43.8 (33.5–62.7)	61.5 (43.8–81.8)	387
≥ 60 years	23.2 (20.8–25.9)	9.9 (7.7–13.0)	16.6 (15.0–17.9)	23.9 (20.9–27.2)	34.7 (30.0–39.3)	50.3 (40.8–68.9)	69.4 (49.6–90.0)	577
Mexican American	14.7 (13.0–16.6)	7.4 (5.6–7.9)	10.3 (8.3–11.8)	15.9 (13.4–17.9)	21.1 (18.7–23.5)	28.1 (24.1–35.0)	35.5 (28.9–38.5)	485
Non-Hispanic black	21.6 (19.1–24.4)	9.9 (7.5–11.9)	14.8 (12.5–16.8)	22.0 (19.5–24.9)	32.2 (28.1–36.2)	43.8 (37.2–57.3)	57.5 (43.8–78.4)	538
Non-Hispanic white	21.4 (19.9–23.1)	10.5 (9.5–11.5)	15.0 (14.4–16.0)	21.9 (20.5–23.0)	30.2 (27.7–33.0)	41.3 (35.7–49.6)	55.9 (44.0–69.4)	962
Female	18.4 (17.0–20.0)	9.0 (7.8–9.9)	12.4 (11.5–13.8)	18.2 (16.8–19.7)	27.3 (23.6–30.0)	39.7 (34.4–42.6)	45.7 (42.3–61.5)	1,041
Male	23.3 (21.1–25.6)	12.3 (10.4–13.5)	17.7 (15.9–18.9)	23.9 (22.3–25.3)	32.1 (28.7–35.7)	45.3 (35.5–62.7)	62.7 (43.8–81.8)	1,053

**Table 3:** Geometric mean and selected percentiles (95% confidence intervals) of perfluorooctanoate (PFOA) concentrations in serum (μg/L) for the U.S. population 12 years of age and older: data from NHANES 2003–2004.

Variable	Geometric mean	10th percentile	25th percentile	50th percentile	75th percentile	90th percentile	95th percentile	No.
All	3.9 (3.6–4.3)	1.9 (1.8–2.1)	2.7 (2.6–3.0)	4.0 (3.8–4.4)	5.8 (5.2–6.3)	7.8 (6.7–9.6)	9.8 (7.4–14.1)	2,094
12–19 years	3.9 (3.5–4.4)	2.2 (1.9–2.3)	2.9 (2.6–3.2)	3.9 (3.3–4.4)	5.4 (4.6–6.1)	6.9 (5.6–9.2)	8.6 (5.9–12.6)	640
20–39 years	3.9 (3.6–4.2)	1.8 (1.5–2.1)	2.7 (2.5–3.0)	4.1 (3.7–4.5)	5.8 (5.4–6.1)	7.6 (7.3–8.4)	9.6 (8.4–11.1)	490
40–59 years	4.2 (3.8–4.8)	2.0 (1.8–2.4)	2.9 (2.6–3.2)	4.2 (3.9–4.8)	6.3 (5.3–7.2)	8.2 (6.8–10.7)	10.6 (7.4–16.9)	387
≥ 60 years	3.7 (3.3–4.1)	1.8 (1.5–2.1)	2.7 (2.4–2.9)	3.9 (3.5–4.3)	5.4 (4.9–5.9)	7.2 (6.0–9.5)	9.5 (6.9–14.1)	577
Mexican American	3.1 (2.8–3.4)	1.4 (1.1–1.8)	2.2 (1.9–2.5)	3.3 (3.0–3.6)	4.4 (4.1–5.1)	6.7 (5.7–7.3)	7.6 (6.7–10.5)	485
Non-Hispanic black	3.4 (3.0–3.8)	1.2 (1.1–1.6)	2.2 (1.9–2.5)	3.7 (3.1–4.2)	5.1 (4.4–6.1)	7.7 (5.3–10.9)	9.3 (6.5–13.9)	538
Non-Hispanic white	4.2 (3.9–4.5)	2.1 (2.0–2.3)	3.0 (2.6–3.2)	4.2 (3.9–4.6)	5.9 (5.4–6.6)	7.8 (7.2–9.1)	9.8 (7.6–13.3)	962
Female	3.5 (3.2–3.8)	1.6 (1.5–1.9)	2.5 (2.2–2.7)	3.6 (3.2–3.9)	5.2 (4.6–5.7)	7.1 (6.3–8.2)	8.4 (7.4–10.6)	1,041
Male	4.5 (4.1–4.9)	2.3 (2.0–2.4)	3.2 (3.1–3.5)	4.6 (4.2–5.0)	6.3 (5.6–7.1)	8.3 (6.8–11.8)	10.4 (7.4–17.5)	1,053

**Table 4:** Geometric mean and selected percentiles (95% confidence intervals) of perfluorohaxanesulfonate (PFHxS) concentrations in serum (μg/L) for the U.S. population 12 years of age and older: data from NHANES 2003–2004.

Variable	Geometric mean	10th percentile	25th percentile	50th percentile	75th percentile	90th percentile	95th percentile	No.
All	1.9 (1.7–2.2)	0.7 (0.6–0.7)	1.0 (0.9–1.2)	1.9 (1.6–2.1)	3.3 (2.8–3.9)	5.9 (4.8–7.2)	8.3 (7.1–9.7)	2,094
12–19 years	2.4 (2.1–2.9)	0.6 (0.5–0.8)	1.2 (1.0–1.4)	2.3 (1.7–3.0)	4.8 (3.9–6.0)	9.5 (6.8–12.5)	13.1 (9.9–19.6)	640
20–39 years	1.8 (1.6–2.0)	0.5 (0.5–0.6)	1.0 (0.9–1.2)	1.7 (1.5–2.0)	2.8 (2.5–3.3)	4.8 (3.9–6.1)	6.7 (4.9–9.4)	490
40–59 years	1.9 (1.6–2.2)	0.7 (0.5–0.8)	1.0 (0.9–1.2)	1.6 (1.4–2.0)	3.1 (2.3–4.5)	5.5 (4.3–6.9)	6.7 (5.5–8.2)	387
≥ 60 years	2.0 (1.7–2.4)	0.8 (0.5–0.9)	1.1 (1.0–1.3)	1.9 (1.6–2.1)	3.2 (2.6–3.7)	7.2 (4.3–9.7)	10.2 (7.0–12.6)	577
Mexican American	1.4 (1.2–1.7)	0.5 (0.3–0.7)	0.7 (0.5–0.9)	1.4 (1.2–1.7)	2.3 (1.9–2.7)	4.2 (3.1–5.1)	5.4 (4.0–8.9)	485
Non-Hispanic black	1.9 (1.6–2.3)	0.5 (0.3–0.7)	1.1 (0.9–1.3)	1.9 (1.5–2.2)	3.4 (2.7–4.3)	6.0 (5.0–7.1)	8.2 (6.3–12.0)	538
Non-Hispanic white	2.0 (1.8–2.3)	0.7 (0.6–0.8)	1.1 (1.0–1.3)	1.9 (1.6–2.1)	3.3 (2.8–4.0)	6.0 (4.6–7.8)	8.1 (6.9–10.1)	962
Female	1.7 (1.6–1.9)	0.6 (0.5–0.6)	0.9 (0.8–1.0)	1.5 (1.4–1.8)	2.9 (2.5–3.5)	5.8 (4.6–6.9)	8.2 (6.7–10.0)	1,041
Male	2.2 (1.9–2.5)	0.8 (0.7–1.0)	1.3 (1.1–1.4)	2.0 (1.8–2.4)	3.3 (2.8–4.4)	6.1 (4.6–8.1)	8.5 (6.4–10.5)	1,053

**Table 5:** Geometric mean and selected percentiles (95% confidence intervals) of perfluorononanoate (PFNA) concentrations in serum (μg/L) for the U.S. population 12 years of age and older: data from NHANES 2003–2004.

Variable	Geometric mean	10th percentile	25th percentile	50th percentile	75th percentile	90th percentile	95th percentile	No.
All	1.0 (0.8–1.1)	0.4 (0.3–0.4)	0.6 (0.5–0.6)	1.0 (0.9–1.1)	1.5 (1.2–1.7)	2.2 (1.6–3.8)	3.2 (1.8–7.7)	2,094
12–19 years	0.9 (0.7–1.0)	0.3 (0.3–0.4)	0.5 (0.5–0.6)	0.7 (0.6–0.9)	1.2 (0.9–1.5)	1.9 (1.2–3.3)	2.7 (1.3–6.3)	640
20–39 years	1.0 (0.8–1.1)	0.3 (0.2–0.5)	0.6 (0.6–0.7)	0.9 (0.8–1.1)	1.4 (1.2–1.7)	2.1 (1.7–2.7)	2.8 (1.9–6.1)	490
40–59 years	1.1 (0.9–1.4)	0.5 (0.4–0.5)	0.7 (0.6–0.7)	1.0 (0.9–1.2)	1.7 (1.2–2.4)	2.7 (1.6–5.9)	4.3 (1.7–9.3)	387
≥ 60 years	0.8 (0.7–1.0)	0.3 (0.2–0.3)	0.5 (0.5–0.6)	0.9 (0.8–1.0)	1.3 (1.1–1.5)	1.9 (1.5–3.0)	3.0 (1.6–6.5)	577
Mexican American	0.7 (0.6–0.8)	0.2 (0.1–0.2)	0.5 (0.4–0.5)	0.7 (0.5–0.8)	1.0 (0.9–1.3)	1.6 (1.2–1.8)	2.0 (1.6–2.8)	485
Non-Hispanic black	1.1 (0.8–1.5)	0.4 (0.3–0.6)	0.6 (0.5–0.8)	1.0 (0.8–1.4)	1.6 (1.2–2.7)	3.1 (1.5–6.5)	4.7 (2.1–9.3)	538
Non-Hispanic white	1.0 (0.8–1.1)	0.4 (0.3–0.4)	0.5 (0.5–0.6)	0.8 (0.8–0.9)	1.5 (1.2–1.7)	2.2 (1.6–3.4)	2.9 (1.8–6.2)	962
Female	0.9 (0.7–1.0)	0.4 (0.3–0.4)	0.6 (0.5–0.6)	0.9 (0.7–0.9)	1.2 (1.0–1.6)	2.2 (1.4–3.3)	3.0 (1.7–6.1)	1,041
Male	1.1 (0.9–1.3)	0.5 (0.4–0.5)	0.6 (0.6–0.7)	1.0 (0.9–1.2)	1.6 (1.3–1.8)	2.4 (1.7–4.8)	4.0 (1.8–8.7)	1,053

**Table 6:** Least-square geometric mean concentrations (μg/L) (95% confidence intervals) of PFOA, PFOS, PFHxS, and PFNA in various demographic groups.

Group	PFOA	PFOS	PFHxS	PFNA
Female		18.5 (17.1–20)		0.9 (0.7–1)
Male		23.6 (21.8–25.7)		1.1 (0.9–1.3)
Female: age P25	3.4 (3.1–3.7)		1.7 (1.5–1.9)	
Female: age P50	3.5 (3.3–3.8)		1.7 (1.5–1.9)	
Female: age P75	3.7 (3.4–4)		1.7 (1.5–2)	
Female: age P90	3.8 (3.4–4.2)		1.7 (1.5–2)	
Male: age P25	5.1 (4.7–5.5)		2.4 (2–2.8)	
Male: age P50	4.5 (4.2–4.9)		2.2 (1.9–2.6)	
Male: age P75	4.1 (3.7–4.5)		2.1 (1.8–2.4)	
Male: age P90	3.7 (3.2–4.2)		1.9 (1.6–2.3)	
MA			1.4 (1.1–1.7)	
NHB			1.9 (1.6–2.3)	
NHW			2.0 (1.8–2.3)	
Female, MA	2.6 (2.3–3)			
Female, NHB	2.8 (2.5–3.2)			
Female, NHW	3.8 (3.5–4.1)			
Male, MA	3.6 (3.3–3.9)			
Male, NHB	4.1 (3.5–4.8)			
Male, NHW	4.6 (4.2–5.1)			
MA: age P25		13.9 (12.5–15.5)		0.7 (0.6–0.8)
MA: age P50		15.1 (13.6–16.8)		0.7 (0.6–0.8)
MA: age P75		16.3 (14.4–18.4)		0.7 (0.5–0.8)
MA: age P90		17.7 (15.3–20.6)		0.6 (0.5–0.8)
NHW: age P25		20.1 (18.6–21.8)		1 (0.8–1.2)
NHW: age P50		21.2 (19.6–22.9)		1 (0.8–1.1)
NHW: age P75		22.3 (20.5–24.3)		0.9 (0.8–1.1)
NHW: age P90		23.5 (21.3–26)		0.9 (0.8–1)
NHB: age P25		19.9 (17.9–22.1)		1.1 (0.8–1.4)
NHB: age P50		22.6 (20.1–25.5)		1.2 (0.9–1.6)
NHB: age P75		25.5 (22.1–29.5)		1.3 (1–1.9)
NHB: age P90		29.0 (24.3–34.7)		1.5 (1–2.1)
NonSMK: age P25				1 (0.8–1.1)
NonSMK: age P50				1 (0.8–1.1)
NonSMK: age P75				1 (0.8–1.1)
NonSMK: age P90				1 (0.8–1.2)
SMK: age P25				1.1 (0.9–1.3)
SMK: age P50				1 (0.8–1.1)
SMK: age P75				0.9 (0.8–1)
SMK: age P90				0.8 (0.7–1)
< HS				0.7 (0.6–0.8)
= HS				1 (0.8–1.1)
> HS				1.2 (0.9–1.7)
< HS: age P25	3.7 (3.4–4.1)			
< HS: age P50	3.7 (3.4–4.1)			
< HS: age P75	3.7 (3.3–4.1)			
< HS: age P90	3.7 (3.3–4.2)			
= HS: age P25	4.4 (4.1–4.7)			
= HS: age P50	4 (3.7–4.3)			
= HS: age P75	3.7 (3.3–4.1)			
= HS: age P90	3.3 (2.8–4)			
> HS: age P25	4.2 (3.8–4.6)			
> HS: age P50	4.1 (3.7–4.5)			
> HS: age P75	4.1 (3.6–4.6)			
> HS: age P90	4 (3.4–4.7)			

Abbreviations: HS, high school; MA, Mexican American; NHB, non-Hispanic black; NHW, non-Hispanic white; NonSMK, nonsmoker; P25, 25th percentile of age = 26 years; P50, 50th percentile of age = 41 years; P75, 75th percentile of age = 55 years; P90, 90th percentile of age = 70 years; SMK, smoker.

**Table 7:** Geometric mean concentrations (95% confidence intervals) of PFOA, PFOS, PFHxS, and PFNA in NHANES 1999–2000 and NHANES 2003–2004 for the whole population and different demographic groups.^a^

	PFOS		PFOA		PFHxS		PFNA	
Variable	1999–2000	2003–2004	1999–2000	2003–2004	1999–2000	2003–2004	1999–2000	2003–2004
All	30.4 (27.1–33.9)	20.7 (19.2–22.3)	5.2 (4.7–5.7)	3.9 (3.6–4.3)	2.1 (1.9–2.4)	1.9 (1.7–2.2)	0.5 (0.5–0.7)	1.0 (0.8–1.1)
12–19 years	29.1 (26.2–32.4)	19.3 (17.5–21.4)	5.5 (5.0–6.0)	3.9 (3.5–4.4)	2.7 (2.1–3.4)	2.4 (2.1–2.9)	0.5 (0.4–0.5)	0.9 (0.7–1.0)
20–39 years	27.5 (24.9–30.2)	18.7 (17.3–20.1)	5.2 (4.7–5.7)	3.9 (3.6–4.2)	2.0 (1.7–2.3)	1.8 (1.6–2.0)	0.5 (0.4–0.6)	1.0 (0.8–1.1)
40–59 years	33.0 (28.0–38.8)	22.0 (19.7–24.5)	5.4 (4.7–6.2)	4.2 (3.8–4.8)	2.1 (1.8–2.3)	1.9 (1.6–2.2)	0.6 (0.4–0.7)	1.1 (0.9–1.4)
≥ 60 years	33.3 (28.5–38.8)	23.2 (20.8–25.9)	4.8 (4.3–5.5)	3.7 (3.3–4.1)	2.2 (1.9–2.5)	2.0 (1.7–2.4)	0.6 (0.5–0.8)	0.8 (0.7–1.0)
Female	28.0 (24.6–31.8)	18.4 (17.0–20.0)	4.8 (4.3–5.3)	3.5 (3.2–3.8)	1.8 (1.6–2.1)	1.7 (1.6–1.9)	0.5 (0.4–0.6)	0.9 (0.7–1.0)
Male	33.4 (29.6–37.6)	23.3 (21.1–25.6)	5.7 (5.2–6.3)	4.5 (4.1–4.9)	2.6 (2.3–3.0)	2.2 (1.9–2.5)	0.6 (0.5–0.7)	1.1 (0.9–1.3)
Mexican American	22.7 (19.8–25.9)	14.7 (13.0–16.6)	3.9 (3.6–4.2)	3.1 (2.8–3.4)	1.5 (1.1–1.9)	1.4 (1.2–1.7)	0.3 (0.3–0.4)	0.7 (0.6–0.8)
Non-Hispanic black	33.0 (26.2–41.6)	21.6 (19.1–24.4)	4.8 (4.1–5.6)	3.4 (3.0–3.8)	2.2 (1.6–2.9)	1.9 (1.6–2.3)	0.8 (0.6–1.0)	1.1 (0.8–1.5)
Non-Hispanic white	32.0 (29.1–35.2)	21.4 (19.9–23.1)	5.6 (5.0–6.2)	4.2 (3.9–4.5)	2.3 (2.0–2.5)	2.0 (1.8–2.3)	0.6 (0.5–0.7)	1.0 (0.8–1.1)

a For PFOS, PFOA, and PFNA, all differences between NHANES 1999–2000 (Calafat et al. 2007) and NHANES 2003–2004 geometric mean concentrations are statistically significant (*p* < 0.001). For PFHxS, except for Mexican Americans (*p* = 0.209), all other differences are also statistically significant with *p* < 0.001, except for females (*p* = 0.037), persons ≥ 60 years of age (*p* = 0.016), persons 12–19 years of age (*p* = 0.004), and non-Hispanic blacks (*p* = 0.004).
